# Preparation of Heat-Resistant Methyl Vinyl Phenyl Silicone Rubber and Study on Its Flexible Strain-Sensing Performance

**DOI:** 10.3390/polym18101149

**Published:** 2026-05-07

**Authors:** Linlin Ouyang, Zhanbo Wang, Depeng Gong, Chaocan Zhang

**Affiliations:** School of Materials Science and Engineering, Wuhan University of Technology, Wuhan 430070, China; oy15070652760@163.com (L.O.); wzb159200@163.com (Z.W.); gdp@whut.edu.cn (D.G.)

**Keywords:** methyl vinyl phenyl silicone rubber, heat resistance, flake silver powder, sandwich-structured sensor

## Abstract

Conventional flexible substrates for strain sensors generally exhibit good flexibility and processability; however, their limited heat resistance restricts their long-term application in high-temperature environments. Aiming at the problem of insufficient heat resistance of high-temperature flexible strain sensing matrix, triphenyltetramethylcyclotrisiloxane (P_3_), trimethyltrivinylcyclotrisiloxane (V_3_) and octamethylcyclotetrasiloxane (D_4_) were used as raw materials in this paper. Methyl vinyl phenyl silica gel (MVMPS) with high phenyl and vinyl content was prepared by anionic ring-opening polymerization, and condensed with KH-570 (3-Methacryloxypropyltrimethoxysilane) to obtain a condensed modified gel (C-MVMPS). Subsequently, a methyl vinyl phenyl silicone rubber composite was fabricated using fumed silica as the reinforcing filler and Si69 as the coupling agent and vulcanization assistant. In addition, flake silver powder was incorporated to prepare an Ag/MVMPS conductive adhesive, and a sandwich-structured strain sensor with a silicone rubber/Ag-MVMPS conductive adhesive/silicone rubber configuration was fabricated. The synthesized methyl vinyl monophenyl silicone gum exhibited a number-average molecular weight of 170,449, a phenyl content of 25.19%, and a vinyl content of 24.44%. The composite showed the best overall performance at 3 phr (parts per hundred of rubber) Si69 (Bis(gamma-triethoxysilylpropyl) tetrasulfide) and 30 phr SiO_2_ (Fumed silica), with a 5% weight-loss temperature (T_5%_) of 367.14 °C and a 10% weight-loss temperature (T_10%_) of 529.6 °C. The prepared sandwich-structured sensor exhibited clear and stable resistance responses within the strain range of 10–80%. The sensitivity increased with increasing strain, and good reproducibility was maintained under different loading rates. Moreover, the sensor still exhibited continuous and distinguishable cyclic responses after 1000 cycles at 20% strain. These results provide an experimental basis and a feasible design strategy for the application of methyl vinyl phenyl silicone rubber in high-temperature flexible strain sensors.

## 1. Introduction

In recent years, the rapid development of flexible electronic devices has attracted considerable interest in flexible materials, which offer unique advantages such as mechanical compliance, light weight, and wear resistance [1,2,3,4]. However, most currently reported flexible strain sensors are still mainly constructed on the basis of conventional flexible substrates, such as polydimethylsiloxane (PDMS) [5], Ecoflex [6], and thermoplastic polyurethane (TPU) [7]. Although these materials exhibit good flexibility and processability, their limited heat resistance restricts their long-term stable operation in high-temperature environments [8]. Conventional methyl silicone rubber, owing to its Si-O-Si backbone structure, exhibits excellent weather resistance, electrical insulation, and resistance to both high and low temperatures, and has therefore been widely used in sealing, insulation, cushioning, and vibration damping applications [9,10,11]. However, the typical service temperature range of conventional silicone rubber is approximately −50 to 230 °C, and its long-term continuous service temperature usually does not exceed 200 °C [12]. Under vacuum conditions, the thermal stability of silicone rubber can approach 300 °C. Nevertheless, at higher temperatures in oxidative environments, pronounced thermo-oxidative aging and rapid oxidative degradation are induced, which further result in the deterioration of mechanical properties and instability of electrical performance [10]. Therefore, the development of silicone rubber-based flexible sensing materials with both flexibility and high-temperature stability has become a key issue to be addressed in the field of high-temperature flexible electronics.

To improve the heat resistance of rubber, extensive studies in recent years have focused on the combined use of side-group design and functional fillers to enhance its high-temperature stability and mechanical strength [13,14,15]. The introduction of phenyl side groups can increase the rigidity of polysiloxane chain segments, strengthen intermolecular interactions, and improve the stability of the material under high-temperature, irradiation, and harsh-medium conditions. In particular, the incorporation of phenyl groups can increase the service temperature of silicone rubber from 200 °C to above 300 °C [16]. Lin et al. [17] synthesized a series of high-molecular-weight copolysiloxanes with different phenyl contents through the ring-opening copolymerization (ROCP) of octamethylcyclotetrasiloxane (D_4_) and tetramethyltetraphenylcyclotetrasiloxane (D_4_^Ph^) catalyzed by P_5_Cl/KOH, and all samples exhibited initial decomposition temperatures above 400 °C. Peter et al. [18] synthesized copolysiloxanes with various phenyl contents by bulk copolymerization of octamethylcyclotetrasiloxane with octaphenylcyclotetrasiloxane (or tetramethyltetraphenylcyclotetrasiloxane), and the phenyl content could reach as high as 50 mol%. The results showed that the thermal stability of the copolysiloxanes increased significantly with increasing phenyl content, and the onset decomposition temperature could reach 519 °C. Sheng et al. [19] reported that, when the filler content was fixed at 40 wt%, the composite containing 20 mol% phenyl groups exhibited a T_5%_ of 454 °C, together with a tensile strength of 3.10 MPa, an elongation at break of 145%, and a hardness of 56 Shore A. Han et al. [20] introduced CeO_2_ and graphene synergistically into a phenyl vinyl methyl silicone rubber (PVMQ) system, increasing the T_5%_ from 329 °C to 438 °C, while achieving a tensile strength of 4.67 MPa and an elongation at break of approximately 180%. Liu et al. [21] synthesized a polysiloxane with a high phenyl content via an alkanol condensation reaction, and further reinforced the modified silicone matrix using high-aspect-ratio needle-shaped silver-plated glass fibers (AGFs) as conductive fillers. The resulting material system exhibited excellent heat resistance, with a T_5%_ of 478 °C, and good mechanical strength, with a tensile strength of 4.67 MPa. Therefore, polysiloxane systems modified synergistically by phenyl groups and fillers can effectively overcome the insufficient heat resistance and mechanical strength of conventional methyl silicone rubber under high-temperature conditions, demonstrating promising application potential in high-performance silicone rubbers and their functional composites [22].

With the expansion of flexible electronics from room-temperature human motion monitoring to high-temperature scenarios, such as temperature monitoring in aerospace vehicles and health monitoring of industrial hot-end equipment, high-temperature-resistant flexible sensors have become an important research direction in flexible devices [23,24,25]. However, most conventional polymer matrix materials are unable to withstand long-term service under high-temperature conditions [26,27]. Liu et al. [28] fabricated a flexible piezoresistive sensor based on a three-dimensional Pt/polyimide (3D Pt/PI) fibrous network by combining electrospinning and ion sputtering, which exhibited excellent sensing performance over a wide temperature range from liquid nitrogen temperature to 250 °C. Jia et al. [29] incorporated multi-walled carbon nanotubes (MWCNTs) into a poly(amic acid ammonium salt) (PAAS) solution as the conductive component and prepared an MWCNT/polyimide multilayer aerogel paper-based sensor that maintained a stable electrical response at 300 °C. Zhou et al. [30] designed a flexible high-temperature-resistant piezoelectric sensor based on cyclized polyacrylonitrile (PAN) and barium titanate (BTO) nanoparticles, which could even realize vibration monitoring at temperatures up to 500 °C. In contrast, most silicone rubber-based flexible sensing materials currently remain limited to strain-responsive applications at room temperature or under low-to-moderate temperature conditions, and their long-term service performance in high-temperature environments still needs to be further improved [31,32,33]. Therefore, the development of silicone rubber-based composites with high-temperature resistance, flexibility, and electrical response capability, as well as their further application in flexible sensing structures for high-temperature environments, is of great theoretical significance and practical value.

Unlike the cyclotetrasiloxane monomers reported in the literature, in this study, methyl vinyl monophenyl silicone rubber (MVMPS) with high phenyl and high vinyl contents was prepared by the copolymerization of triphenyltrimethylcyclotrisiloxane (P_3_), trivinylcyclotrisiloxane (V_3_), and octamethylcyclotetrasiloxane. The introduction of phenyl groups contributes to improving the thermal stability of silicone rubber, while vinyl groups provide reactive sites for subsequent crosslinking. In addition, bis[3-(triethoxysilyl)propyl] tetrasulfide (Si69) was employed as the primary vulcanizing agent to construct the crosslinked network. The triethoxysilyl groups in Si69 can undergo hydrolysis–condensation reactions with silanol groups on the surface of silica, thereby strengthening the interfacial interaction between the filler and the rubber matrix and improving filler dispersion. The effects of Si69 and SiO_2_ contents were systematically investigated, and the optimal formulation for this composite system was determined. Furthermore, flake silver powder was introduced to fabricate Ag/MVMPSD conductive silicone composites. A sandwich-structured strain sensor consisting of “phenyl silicone rubber-Ag/MVMPS conductive layer-phenyl silicone rubber” was then constructed, and the feasibility of this composite system as a high-temperature-resistant flexible strain-sensing matrix was preliminarily explored.

## 2. Materials and Methods

### 2.1. Materials

Octamethylcyclotetrasiloxane (D_4_, C_8_H_24_O_4_Si_4_, 98% purity) was purchased from Aolilong Chemical Co., Ltd., Jining, China. Trimethyltrivinylcyclotrisiloxane (V_3_, 98% purity) was purchased from Hubei Changfu Chemical Co., Ltd., Wuhan, China. Triphenyltetramethylcyclotrisiloxane (P_3_, C_48_H_40_O_4_Si_4_, 98% purity) was purchased from Zhejiang Xinan Chemical Industrial Group Co., Ltd., Hangzhou, China. Tetramethylammonium hydroxide (TMAOH, C_4_H_13_NO, 25wt% aqueous solution) was purchased from Shanghai Macklin Biochemical Co., Ltd., Shanghai, China. 3-Methacryloxypropyltrimethoxysilane (KH-570, 98% purity) was purchased from Dongguan Kangjin New Material Technology Co., Ltd., Dongguan, China. Dibutyltin dilaurate (95% purity) was purchased from Shanghai Macklin Biochemical Co., Ltd., Shanghai, China. Fumed silica (SiO_2_, HB-630, particle size(7–40 nm), BET surface area(270 ± 30 m^2^/g)) was supplied by Hubei Huifu Nanomaterials Co., Ltd., Yichang, China. Dicumyl peroxide (DCP, industrial grade) was purchased from Hubei Langbowan Biopharmaceutical Co., Ltd., Huanggang, China. 2,5-Dimethyl-2,5-di(tert-butylperoxy)hexane (DHBP-50B) was purchased from Changsha Zhongyi Chemical Co., Ltd., Changsha, China. Bis(gamma-triethoxysilylpropyl) tetrasulfide (Si69, 98% purity) was purchased from Dongguan Kangjin New Material Technology Co., Ltd., Dongguan, China. Flake silver powder (research grade, particle size (1000 mesh)) was purchased from Shanghai Xinzhuan Alloy Materials Co., Ltd., Shanghai, China.

### 2.2. Synthesis of the Catalyst Masterbatch

To improve the compatibility between the initiator and the monomers, a catalyst masterbatch was prepared using D_4_ and TMAOH as the starting materials. A portion of D_4_ and TMAOH was added to a flask equipped with a nitrogen inlet, a mechanical stirrer, a condenser, and a thermometer. The stirring speed was controlled within an appropriate range, and the temperature was raised to 50 °C. The system was then evacuated at −0.09 MPa for 40 min to remove water, after which the vacuum pump was turned off, and nitrogen was introduced to restore atmospheric pressure. Subsequently, the reaction system was heated to 80 °C and maintained at this temperature for 2 h, affording a transparent and viscous catalyst masterbatch with high activity.

### 2.3. Synthesis of Methyl Vinyl Monophenyl Silicone Gum (MVMPS)

**Figure 1:**
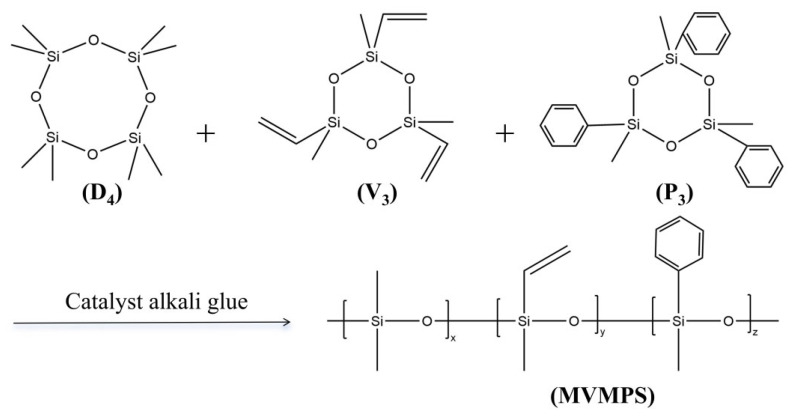
The synthetic route of MVMPS.

A predetermined amount of D_4_ and V_3_ was added to a three-necked flask. The system temperature was raised to 60 °C, and the pressure was reduced to −0.09 MPa under vacuum. The stirring speed was set at 150 r/min, and dehydration was carried out for 40 min. The system was then returned to atmospheric pressure by introducing nitrogen. Subsequently, the weighed P_3_ was added, and dehydration was continued for another 1 h. Thereafter, the prepared catalyst masterbatch was added, and the system was adjusted to the same conditions as described above for a further dehydration period of approximately 1 h. After dehydration, the vacuum was released, and nitrogen was introduced to restore atmospheric pressure. Under nitrogen protection, the reaction was conducted at 110 °C for 3 h with an appropriate stirring speed and nitrogen bubbling rate. As the temperature increased, the catalyst activity was gradually destroyed, and the catalyst decomposed into trimethylamine and methanol. Finally, trimethylamine, methanol, and some unreacted monomers were removed under vacuum at 170 °C. The synthetic route of MVMPS is illustrated in Figure 1.

### 2.4. Condensation Modification of MVMPS

The MVMPS raw gum was first added into an internal mixer and plasticated at an appropriate rotational speed until it became semi-transparent, so as to ensure smooth feeding during the subsequent process. Then, an appropriate amount of dibutyltin dilaurate (DBTDL) and 3-methacryloxypropyltrimethoxysilane (KH-570) was added, and the temperature was raised to 80 °C and maintained for a certain period to ensure uniform mixing of all components. After complete mixing, the temperature was increased to 90 °C to carry out the chain-extension reaction for 40 min, yielding the condensation-modified gum (C-MVMPS).

The reaction can be described as follows. The hydroxyl-terminated methyl vinyl phenyl silicone gum was denoted as HO-SiP-OH, where P represents the siloxane backbone segment substituted with methyl, vinyl, and phenyl groups. KH-570 was denoted as (MeO)_3_Si-R, where R is (CH_2_)_3_OCOC(CH_3_)=CH_2_. Under DBTDL catalysis and elevated temperature, the terminal hydroxyl groups of the silicone chain condensed with the methoxy groups of KH-570, as shown in Formula (1):

(1) $$\begin{array}{c} HO-SiP-OH+{\left(MeO\right)}_{3}Si-R\to \\ HO-SiP-O-{\left(MeO\right)}_{2}Si-R+MeOH \end{array}$$

This was followed by further stepwise condensation, as shown in Formula (2):

(2) $$HO-SiP-OH+HO-SiP-O-{\left(MeO\right)}_{2}Si-R\to \;\begin{array}{c} HO-SiP-O-\left(MeO\right)Si-R-O-SiP-OH+MeOH \end{array}$$

### 2.5. Preparation of SiO_2_/MVMPS Composites

The basic formulation consisted of 100 phr C-MVMPS, different amounts of fumed silica, 2 phr hydroxyl silicone oil, and Si69. First, the raw gum was added into the internal mixer and plasticated at an appropriate rotational speed until it became semi-transparent to ensure smooth feeding. After the temperature was raised to 90 °C, Si69, different amounts of fumed silica, and the structure control agent were added sequentially. The fumed silica was added in batches to ensure its sufficient and uniform dispersion in the matrix. After mixing, the compound was removed from the internal mixer and allowed to stand at room temperature for 12 h to cool and to ensure sufficient interaction among all components. The compound was then subjected to two-stage vulcanization. The first-stage vulcanization was carried out on a plate vulcanization instrument, and the vulcanization conditions were set to 170 °C, 10 MPa, and about 23 min. The second-stage vulcanization was performed in an oven at 190 °C for 2 h. Finally, the methyl vinyl monophenyl silicone rubber composite was obtained.

### 2.6. Preparation of Ag/MVMPS Conductive Adhesive

The C-MVMPS compound was plasticated in an internal mixer until it became semi-transparent. Flake silver powder was then added in batches at 90 °C under an appropriate rotational speed, and each addition was thoroughly mixed to ensure uniform dispersion. Subsequently, Si69 and the structure control agent were added sequentially. After mixing, the compound was removed from the internal mixer and allowed to stand at room temperature for 12 h to ensure sufficient interaction among the components. Vulcanization was carried out on a plate vulcanization instrument, and the vulcanization conditions were set to 170 °C, 10 MPa, and about 23 min.

### 2.7. Preparation of the Phenyl Silicone Rubber/Conductive Adhesive/Phenyl Silicone Rubber Sandwich-Structured Sensor

**Figure 2:**
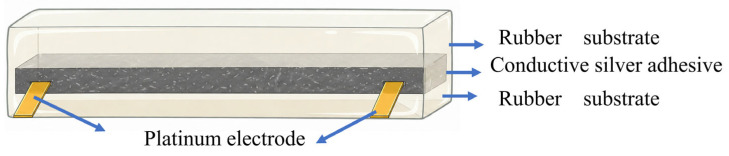
Schematic diagram of a sandwich structure.

Methyl vinyl phenyl silicone rubber was prepared according to the procedure described in Section 2.4, except that only partial vulcanization was conducted on the plate vulcanizing press. Subsequently, the Ag/MVMPS compound prepared in Section 2.6 was placed between two silicone rubber layers and assembled into a mold. Electrodes (platinum electrode) were introduced at both ends, and the assembly was allowed to stand for one day. Co-vulcanization was then performed on the plate vulcanizing press to obtain a silicone rubber/Ag-MVMPS conductive adhesive/silicone rubber sandwich-structured sensor. A sandwich structure sensor was prepared by step-by-step assembly and co-vulcanization. The silver/silicone rubber conductive layer was embedded between the rubber layers and solidified together to form an integrated flexible structure [34]. The sandwich structure is shown in Figure 2; each layer is made of a mold thickness of 1 mm, and 1 mm is the design thickness.

### 2.8. Characterization

Fourier transform infrared (FTIR) spectra were recorded using a Nicolet 6700 Fourier transform infrared spectrometer over the range of 4000–400 cm^−1^ with a resolution of 4 cm^−1^. Proton nuclear magnetic resonance (^1^H-NMR) spectra were recorded on a Bruker AVANCE III HD 500 NMR spectrometer. The molecular weight and molecular weight distribution of the samples were determined using an Agilent 1260 Infinity II system at 40 °C, with tetrahydrofuran (THF) as the mobile phase at a flow rate of 1.0 mL/min. Thermogravimetric analysis (TGA) was carried out using a TGA 8000+Spectrum 3 thermal analyzer. The samples were heated from 30 °C to 1000 °C at a heating rate of 10 °C/min under a nitrogen atmosphere to evaluate their thermal stability and high-temperature decomposition behavior. The dynamic mechanical properties of the vulcanized rubber were measured using a TA Q800 dynamic mechanical analyzer. Dynamic thermomechanical analysis was conducted at a frequency of 10 Hz over the temperature range of −130 to 100 °C with a heating rate of 3 °C/min. Strain sweep measurements of the uncured phenyl silicone rubber were performed using a rubber process analyzer (RPA-8000) at 60 °C and 1 Hz over a strain range of 0.1–400%. The vulcanization characteristics of the rubber materials at 170 °C were measured using an M-3000A rheometer. The tensile strength and elongation at break of the vulcanized samples were tested at 25 °C using a universal testing machine at a tensile rate of 50 mm/min. The hardness was measured using a Shore A durometer (LX-A). The crosslinking density of the phenyl silicone rubber materials in this study was determined by the equilibrium swelling method using toluene as the solvent. The morphology of the samples was observed using a TESCAN MIRA LMS scanning electron microscope (SEM). The electrical conductivity was measured using a four-probe resistivity tester. The sensing performance was evaluated using a tensile-compression testing machine (WDW-L02) combined with a benchtop digital multimeter (HT8808A). The strain loading is applied by the tensile tester, and the electrical output records the resistance change in real time through the desktop digital multimeter.

## 3. Results

### 3.1. Synthesis and Characterization of Methyl Vinyl Monophenyl Silicone Gum (MVMPS)

#### 3.1.1. Synthesis of MVMPS

The anionic ring-opening polymerization of cyclosiloxanes is generally carried out under alkaline conditions. In this study, D_4_, V_3_, and P_3_ were used as cyclic siloxane monomers to synthesize methyl vinyl monophenyl silicone gum through an anionic ring-opening polymerization route. Three different catalysts, namely sodium ethoxide, stannous octoate, and tetramethylammonium hydroxide (TMAOH), were investigated. Under the same reaction conditions, the molecular weights of the products obtained with different catalysts are listed in Table 1.

During the actual experimental process, it was observed that sodium ethoxide was highly sensitive to trace amounts of moisture and impurities in the system, which made the reaction more susceptible to fluctuations and resulted in relatively poor system stability. In contrast, stannous octoate showed limited ability to promote the ring-opening polymerization in this system, leading to a relatively slow reaction rate and making it difficult to achieve a high molecular weight within a reasonable reaction time. By comparison, the tetramethylammonium hydroxide (TMAOH) system exhibited a smoother polymerization initiation process and more efficient chain-growth propagation, ultimately affording products with higher molecular weights and better reproducibility. In addition, the catalyst residue could be more effectively reduced during post-treatment. Therefore, based on the above comparison, tetramethylammonium hydroxide was finally selected as the catalyst for this anionic ring-opening polymerization system.

#### 3.1.2. Structural Characterization of MVMPS

**Figure 3:**
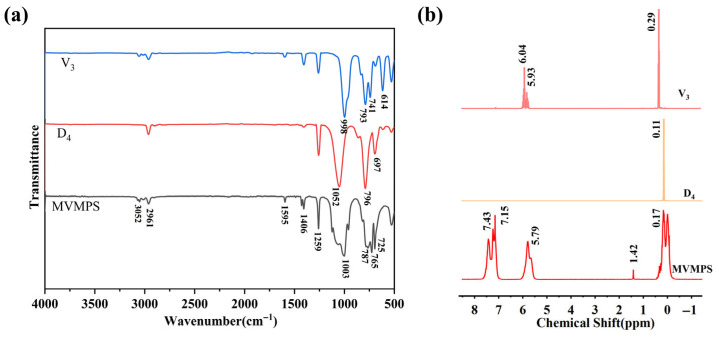
MVMPS and D_4_, V_3_: (**a**) Fourier transform infrared spectroscopy, (**b**) nuclear magnetic resonance hydrogen spectrum.

To investigate the structure of the synthesized product, Fourier transform infrared (FTIR) spectroscopy and proton nuclear magnetic resonance (^1^H-NMR) spectroscopy were performed. The FTIR spectra and ^1^H-NMR spectra of MVMPS, D_4_, and V_3_ are shown in Figure 3. As shown in Figure 3a, the absorption band at around 1003 cm^−1^ was mainly attributed to the asymmetric stretching vibration of the Si-O-Si bond, while the peak near 1259 cm^−1^ corresponded to the symmetric deformation vibration of Si-CH_3_. Compared with D_4_, the product exhibited characteristic absorption peaks associated with aromatic groups at approximately 3050, 1595, 725, and 765 cm^−1^. Compared with V_3_, the product showed an enhanced vibration band of the aromatic ring skeleton in the region around 1595 cm^−1^, together with characteristic out-of-plane bending vibration peaks of aromatic C-H near 725 and 765 cm^−1^, indicating that the phenyl structure from P_3_ had been successfully introduced into the molecular chains of the product. Meanwhile, the absorption bands associated with vinyl groups were still present, indicating that the vinyl side groups were retained after polymerization.

To further verify the molecular structure of the product, the raw monomers and the ring-opening copolymerization product were characterized by ^1^H-NMR spectroscopy, as shown in Figure 3b. The spectrum of the D_4_ monomer showed a strong absorption peak near 0 ppm, corresponding to the methyl proton signal of Si-CH_3_, indicating that its structure mainly consisted of methyl-substituted siloxane units. The V_3_ monomer exhibited a distinct characteristic peak at around 5.9–6.0 ppm, which could be assigned to the resonance signal of vinyl protons (-CH=CH_2_). At the same time, a characteristic Si-CH_3_ absorption was also observed near 0 ppm, indicating that the V_3_ molecule contained both vinyl- and methyl-substituted siloxane structural units. In the ^1^H-NMR spectrum of MVMPS, clear aromatic proton peaks appeared in the range of 7.0–7.5 ppm, vinyl proton signals were observed at approximately 5.7–6.2 ppm, and strong Si-CH_3_ absorption peaks were still retained in the region of 0–0.3 ppm. Compared with the spectra of the monomers, the product simultaneously retained the characteristic proton signals of both phenyl and vinyl groups, indicating that the vinyl and phenyl side groups were successfully introduced into the polysiloxane backbone during the ring-opening polymerization process. Combined with the FTIR and ^1^H-NMR analyses, these results preliminarily confirm the successful synthesis of the target methyl vinyl monophenyl silicone gum.

According to the integrated peak areas of the characteristic proton signals corresponding to different functional groups in the ^1^H-NMR spectrum of MVMPS shown in Figure 3b, the actual phenyl content and vinyl content of the synthesized phenyl silicone rubber can be calculated. The calculated contents of the phenyl and vinyl groups in MVMPS are listed in Table 2.

### 3.2. Structural Characterization of End-Group Condensation-Modified Methyl Vinyl Monophenyl Silicone Gum (C-MVMPS)

**Figure 4:**
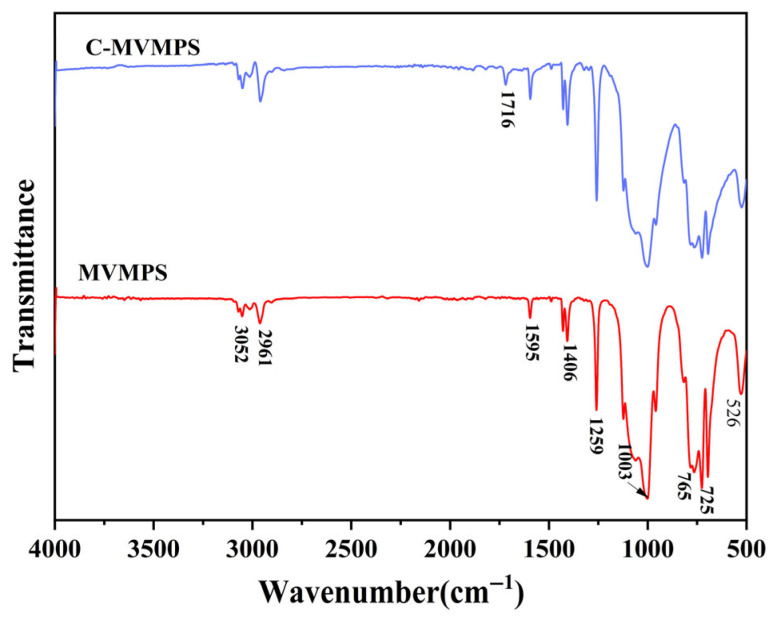
Infrared spectra of MVMPS and C-MVMPS.

Since the obtained raw gum still contained hydroxyl groups at both chain ends, 3-methacryloxypropyltrimethoxysilane (KH-570) was used to carry out condensation modification of the synthesized MVMPS in order to improve the stability of the subsequent system, thereby yielding C-MVMPS. Figure 4 shows the FTIR spectra of MVMPS and C-MVMPS. Compared with the raw gum, the FTIR spectrum of C-MVMPS exhibited new characteristic changes. A distinct absorption peak appeared near 1716 cm^−1^, which was assigned to the C=O stretching vibration of the methacrylate group in KH-570, indicating that KH-570 was successfully introduced into the system. In the range of 1000–1100 cm^−1^, the Si-O-Si absorption band became stronger and showed an obvious change in peak shape, suggesting that a condensation reaction occurred between the hydroxyl-terminated siloxane chains and the -Si(OCH_3_)_3_ groups of KH-570, resulting in the formation of new Si-O-Si bonds.

**Figure 5:**
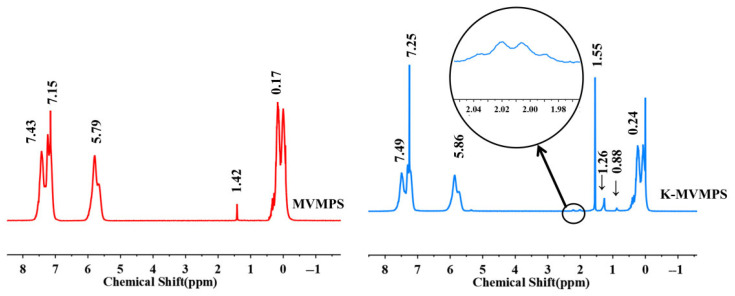
^1^H-NMR spectra of MVMPS and C-MVMPS.

Figure 5 shows the 1H NMR spectra of MVMPS and C-MVMPS. After condensation modification, the spectrum still retained the three main characteristic signals of methyl, vinyl, and phenyl groups, indicating that the siloxane backbone structure was not destroyed. The signals at 1.26 and 0.88 ppm were consistent with the methylene proton signals of the propyl chain (-Si-(CH_2_)_3_-) in KH-570, whereas the signal near 2.00 ppm was assigned to the characteristic resonance of =C-CH_3_ in the methacrylate group. These spectral features are in good agreement with the introduction of the propyl chain and methacrylate group from KH-570, confirming that KH-570 was successfully grafted onto the siloxane chains through terminal hydroxyl condensation.

### 3.3. Investigation of the Vulcanization System

Dicumyl peroxide (DCP) and 2,5-dimethyl-2,5-di(tert-butylperoxy)hexane (DHBP) are important organic peroxide curing agents that are widely used in rubber vulcanization [35]. In the present study, phenyl and vinyl units were introduced into the silicone rubber chains; therefore, different vulcanization systems were preliminarily investigated. The mechanical properties of the MVMPS composites are summarized in Table 3 and Table 4. Specifically, 100 phr of the above-prepared C-MVMPS, together with the same amount of fumed silica, was compounded with different curing agents at different loadings to compare their effects on the material properties. As shown in the tables, both peroxide curing agents exhibited clear incompatibility with the present formulation. At low dosages, the rubber became hard after primary vulcanization and showed a relatively low elongation at break, below 80%. With increasing dosage, the samples became brittle and prone to cracking. This behavior may be attributed to the fact that free radicals generated by the thermal decomposition of peroxide curing agents induced multi-site crosslinking. Owing to the high contents of vinyl and phenyl groups in this system, the radical reaction more readily produced short-range crosslinking, resulting in non-uniform crosslinking and local over-crosslinking. Consequently, the resulting crosslinked network became heterogeneous, leading to stress concentration and an increased tendency toward brittle fracture.

In view of the incompatibility of peroxide curing agents, bis(gamma-triethoxysilylpropyl) tetrasulfide (Si69) was further investigated, and the mechanical properties of the MVMPS composites containing different amounts of Si69 were compared. Si69 is a typical bifunctional silane coupling agent. On the one hand, it can undergo condensation with silanol groups on the surface of silica, thereby improving the compatibility between fumed silica and the rubber matrix and reducing defect sources caused by filler agglomeration [36]. On the other hand, it can act as an alternative vulcanization system, in which its sulfur-containing structure participates in crosslinking, thus improving network uniformity and elongation at break. Therefore, Si69 plays a dual role in this system, namely, interfacial modification and crosslinking. As shown in Table 4, the use of Si69 effectively alleviated the brittleness and cracking observed in the peroxide-cured samples, while also improving the strength and toughness of the phenyl silicone rubber.

### 3.4. Effect of Si69 Content on the Processability of MVMPS Composites

**Figure 6:**
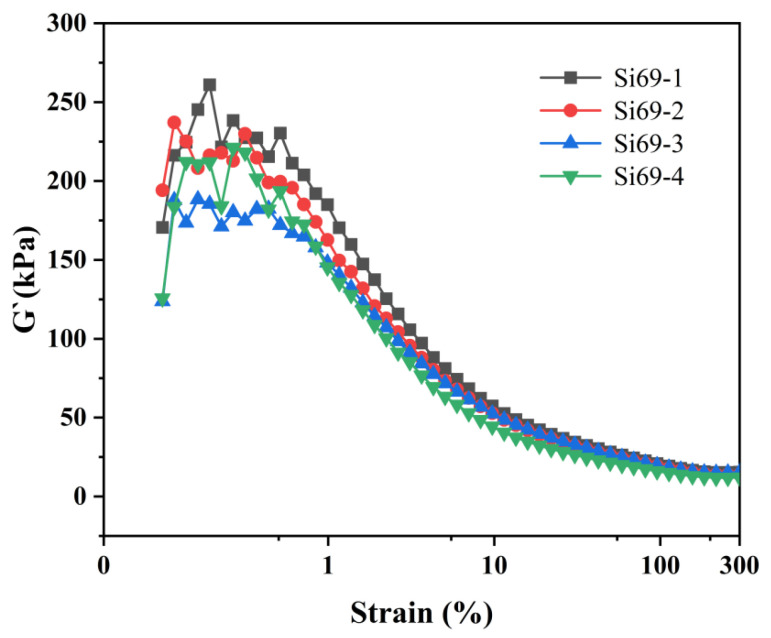
Strain amplitude dependence of MVMPS composites with different Si69 content.

The processing behavior of methyl vinyl phenyl silicone rubber with different Si69 contents is shown in Figure 6. For all compounds, the storage modulus (G′) gradually decreased from the low-strain region (approximately 0.2–1%) to the high-strain region (>10% to 300%) and then tended to level off, which is a typical Payne effect [37]. In the low-strain region, the G′ value of Si69-1 was significantly higher than that of the other groups, indicating that the filler-filler network was the strongest, the degree of agglomeration was more pronounced, and the filler dispersion was relatively poor. By contrast, Si69-3 exhibited a lower G′ value in the low-strain region, suggesting a weaker filler network, better filler dispersion, and thus better processing stability. This can be attributed to the role of Si69 as a silane coupling agent, which reacts with or covers the -SiOH groups on the SiO_2_ surface, thereby reducing hydrogen-bonding interactions and the tendency for secondary agglomeration between filler particles. As a result, the filler network is weakened, and the filler dispersion is improved. In this composite system, fumed silica particles act as physical reinforcing nodes, while Si69 serves as an interfacial coupling bridge between the inorganic filler surface and the silicone rubber molecular chains. The ethoxy groups in Si69 can react with silanol groups on the silica surface, thereby weakening filler-filler hydrogen bonding and improving the dispersion of silica in the silicone rubber matrix [38]. At the same time, the sulfur-containing segments of Si69 can participate in the crosslinking reaction, promoting the formation of more stable interfacial bonding between rubber chains and the filler surface. Therefore, the effective reinforcing network in the composite can be understood as being jointly composed of chemically crosslinked siloxane chains, physically adsorbed chains constrained near the silica surface, and interfacial-bound rubber mediated by Si69.

### 3.5. Effects of Si69 and SiO_2_ Contents on the Vulcanization Characteristics and Crosslinking Density of MVMPS Composites

The vulcanization process of silicone rubber has a significant influence on the physical and mechanical properties of the final molded materials. The vulcanization characteristics of the compounds were measured at 170 °C using a rotorless rheometer, and the results are summarized in Table 5. As shown in the table, the scorch time (t_c10_) of all samples was approximately 90 s, while the optimum cure time (t_c90_) was around 23 min, with a fluctuation of less than 1 min. In addition, the maximum torque (M_H_) increased significantly with increasing Si69 content, and the torque difference (ΔM) showed a pronounced rise starting from the Si69-3 formulation. Notably, the ΔM value of Si69-2 was slightly lower than that of Si69-1 (1.53 < 1.69), which may be attributed to the fact that the interfacial reaction was still insufficient at this stage, while a slight “lubrication/dilution” effect may also have occurred, resulting in a decrease rather than an increase in ΔM. With increasing fumed silica content, the minimum torque (M_L_) remained almost unchanged (0.7–0.8), whereas M_H_ and ΔM increased steadily. However, when the SiO_2_ content reached 40 phr, M_L_, M_H_, and ΔM all increased significantly. This behavior may be explained as follows. At low to medium filler loadings (10–30 phr), the system was mainly dominated by reinforcement and an increase in the bound rubber layer. The addition of fumed silica enhanced the reinforcing effect and generated more constrained rubber chains, thereby increasing the stiffness of the vulcanized system and leading to higher M_H_ and ΔM values. In contrast, when the filler content reached 40 phr, a very strong filler network had already formed in the uncured compound, and the material exhibited obvious structural build-up and yield-like behavior, resulting in a substantial decrease in flowability and processability.

**Figure 7:**
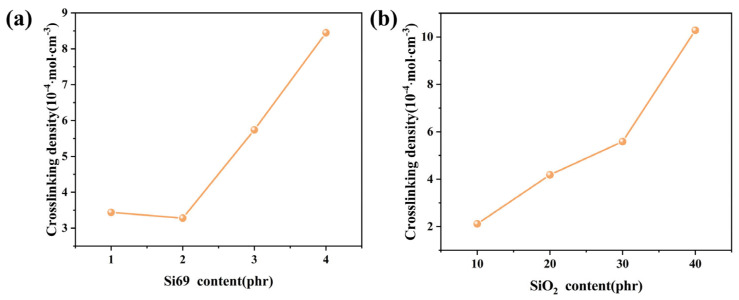
Crosslinking density of MVMPS composites: (**a**) different Si69 contents; (**b**) different SiO_2_ contents.

Figure 7 shows the crosslinking density of methyl vinyl phenyl silicone rubber with different Si69 and SiO_2_ contents. Since the torque difference (ΔM) is generally positively correlated with crosslinking density [39], the trend observed in Figure 7 is consistent with the variation in ΔM. At low Si69 loading, the coupling agent may be insufficient to cover the silica surface, and most silica particles therefore act only as physical fillers. As a result, the interaction between the silicone rubber and filler remains relatively weak, leading to a relatively high swelling degree and thus a low apparent crosslinking density. Hydrophobic silica itself does not absorb solvent; therefore, with increasing silica content, the volume fraction of the rubber phase in the overall material decreases. During the swelling experiment, the filler restricts the extension of rubber molecular chains and hinders solvent penetration, thereby reducing the swelling degree. After the addition of Si69, increasing the silica content provides more reactive sites, i.e., hydroxyl groups on the silica surface, for the coupling reaction. If the amount of Si69 is sufficient to cover the newly introduced silica surface, more inorganic filler particles can be chemically bonded to the silicone rubber matrix, which markedly increases the effective network crosslinking density.

### 3.6. Effects of Si69 and SiO_2_ Contents on the Mechanical Properties of MVMPS Composites

**Figure 8:**
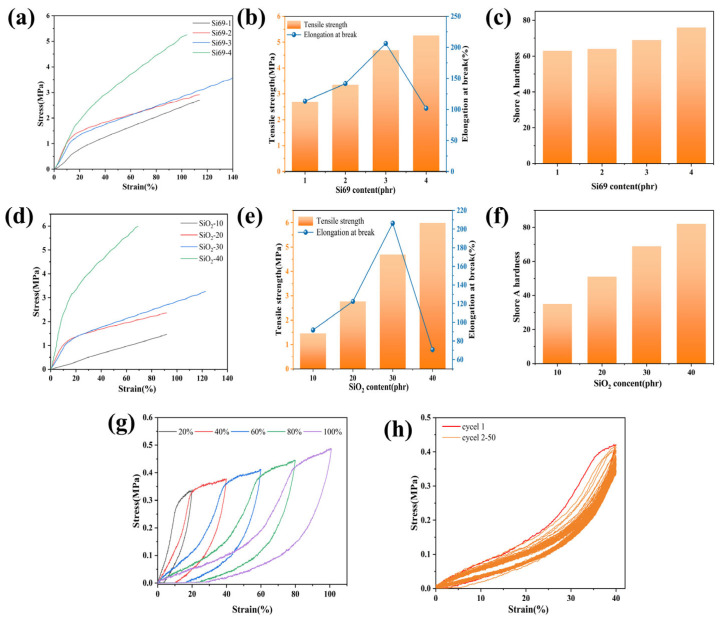
(**a**) Stress–strain curves of the samples with different Si69 contents; (**b**) tensile strength and elongation at break of the samples with different Si69 contents; (**c**) hardness of the samples with different Si69 contents; (**d**) stress-strain curves of the samples with different SiO_2_ contents; (**e**) tensile strength and elongation at break of the samples with different SiO_2_ contents; (**f**) hardness of the samples with different SiO_2_ contents; (**g**) the cyclic stress-strain curve of MVMPS in the range of 20–100% strain; (**h**) the cyclic curves of MVMPS in the first and second-50 cycles of 40% strain stretching.

Based on the preliminary results shown in Table 4, the effects of different Si69 and SiO2 contents on the mechanical properties of the rubber were further investigated by adjusting their formulations. The effects of different Si69 and SiO_2_ contents on the static mechanical properties of the MVMPS composites are shown in Figure 8a-f. As shown in Figure 8a, the stress-strain curves of the samples generally shifted upward with increasing Si69 content. Further combined with the changes in tensile strength and tensile strength retention shown in Figure 8b, it can be seen that the tensile strength generally increased with increasing Si69 content, whereas the elongation at break first increased and then decreased. Specifically, the elongation at break reached its maximum value of 206.28% at 3 phr Si69, and then decreased significantly when the Si69 content was further increased to 4 phr. This phenomenon may be attributed to the fact that excessive Si69 leads to an excessively high local crosslinking density in the system, thereby restricting chain-segment motion and reducing the toughness of the material, which is ultimately reflected by a lower elongation at break.

As shown in Figure 8d, the effect of different SiO_2_ contents on the static mechanical properties of the MVMPS composites exhibited a trend similar to that observed for Si69. When the SiO_2_ content was 30 phr, the retention reached the highest value. When the SiO_2_ content was further increased, the distance between filler particles decreased, which facilitated the formation of agglomerates or a more continuous filler network. This induced stress concentration, making the material more susceptible to brittle failure or rapid fracture during stretching. As a consequence, the strength increased, whereas the elongation at break decreased. As can be seen from Figure 8c,f, the hardness gradually increased with increasing Si69 and SiO_2_ contents. Figure 8g shows the cyclic stress-strain curves of the material under different strains of 20–100%. As the maximum strain increases from 20% to 100%, the hysteresis loop between the loading-unloading curves gradually becomes larger, indicating that the material has obvious energy dissipation and recovery hysteresis during the tensile and recovery processes. The material was subjected to 50 cyclic tensile tests at 40% strain, and the results are shown in Figure 8h. After the first tensile cycle, the hysteresis curves of the subsequent 2–50 cycles gradually tend to overlap, indicating that the material has good fatigue resistance.

**Figure 9:**
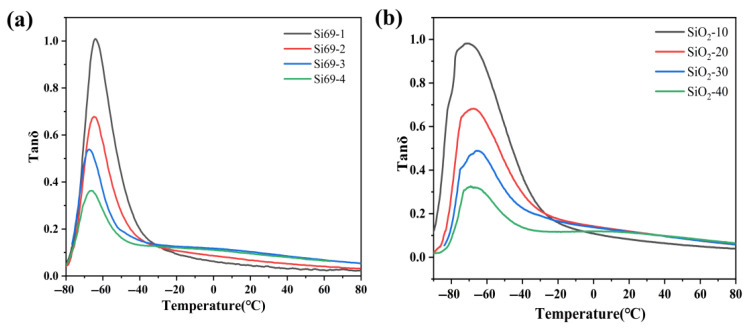
(**a**) DMA curves of the samples with different Si69 contents; (**b**) DMA curves of the samples with different SiO_2_ contents.

To further clarify the effects of Si69 and SiO_2_ contents on the overall properties of the materials, dynamic mechanical analysis (DMA) was performed to investigate the mechanical behavior of the rubber materials under different temperature conditions, as shown in Figure 9. Under essentially unchanged basic formulation conditions, the glass transition temperatures of the composites prepared with different Si69 and SiO_2_ contents showed no significant variation, and all samples exhibited a distinct tan delta peak in the glass transition region. By comparison, it can be seen that the tan delta peak intensity gradually decreased with increasing Si69 and SiO_2_ contents. This trend can be attributed to the enhanced interfacial interaction and increased crosslinking/constraining effect caused by the addition of Si69 and SiO_2_, which weakened the cooperative segmental relaxation during the glass transition process.

### 3.7. Effects of Si69 and SiO_2_ Contents on the Heat Resistance of MVMPS Composites

**Figure 10:**
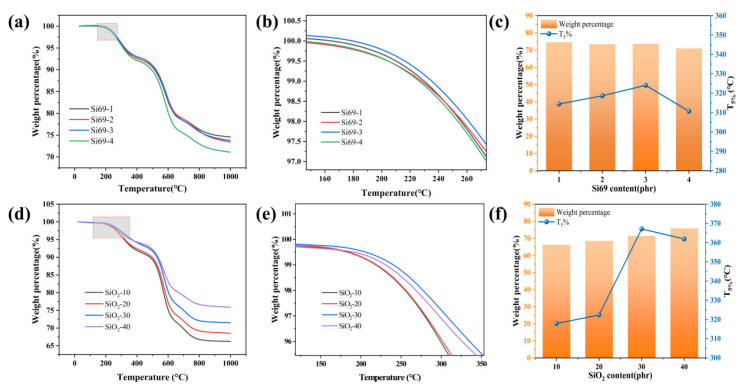
(**a**) TG curves of the samples with different Si69 contents; (**b**) enlarged views of the initial temperature region for the samples with different Si69 contents(the box area in (**a**)); (**c**) T_5%_ values and high-temperature char residues of the samples with different Si69 contents; (**d**) TG curves of the samples with different SiO_2_ contents; (**e**) enlarged views of the initial temperature region for the samples with different SiO_2_ contents (the box area in (**d**)); (**f**) T_5%_ values and high-temperature char residues of the samples with different SiO_2_ contents.

Under high-temperature conditions, electronic devices in long-term operation are often subjected to thermal stress as high as 200 °C or even higher. To meet the stringent requirements for material stability and reliability in such harsh environments, a systematic investigation of the heat resistance of silicone rubber is particularly important. To quantitatively evaluate the thermal decomposition behavior of the methyl vinyl phenyl silicone rubber composite system, the TGA curves, enlarged views of the initial decomposition region, T_5%_ values, and char residues of the samples with different Si69 and SiO_2_ contents are presented in Figure 10a-f. As shown in Figure 10a–c, when the Si69 content increased from a low to a moderate level, the T_5%_ of the samples generally increased. Among them, the sample containing 3 phr Si69 exhibited the highest T_5%_, and its T_10%_ reached 521.83 °C. However, when the Si69 content was further increased to 4 phr, the T_5%_ decreased instead. A possible reason is that excessive Si69 led to locally over-dense structures, which were unfavorable for further improvement in thermal stability. Meanwhile, the differences in char residue among the samples with different formulations were relatively small in the high-temperature region. As shown in Figure 10d-f, with the increase in SiO_2_ content from 10 phr to 40 phr, the thermal decomposition residue of the material continuously increased from 66.21% to 75.86%, showing a monotonic upward trend. This behavior mainly originated from the thermal inertness of inorganic SiO_2_ and its skeleton-supporting effect during thermal decomposition. The T_5%_ reached its highest value at 30 phr SiO_2_ (367.14 °C), while the T_10%_ reached 529.6 °C, indicating that an appropriate increase in SiO_2_ content could improve the T_5%_ and delay the initial stage of thermal decomposition to some extent. However, when the SiO_2_ content was further increased to 40 phr, the T_5%_ decreased to 361.91 °C. This may be attributed to filler agglomeration, increased interfacial defects, and volatilization of low-molecular-weight species at high filler loading. The thermal conductivity of SiO_2_ at room temperature is 1.1–1.2 W/(m·K), which is low. However, when SiO_2_ nanoparticles form a continuous chain, a highly conducting backbone may be formed, triggering a heat transfer at higher SiO_2_ content, thus T_5%_ decreased to 361.91 °C due to low-molecular-weight species loss. Overall, the formulation containing 3 phr Si69 and 30 phr fumed silica achieved a better balance among reinforcing efficiency, toughness, and thermal stability.

Compared with methyl substituents, phenyl groups possess greater steric hindrance and higher structural rigidity, which can effectively restrict the rotational freedom of the Si-O-Si main chain and reduce the mobility of siloxane chain segments under high-temperature conditions [40]. In addition, the aromatic ring structure helps enhance intermolecular interactions among polysiloxane chains, thereby increasing the energy required for chain slippage and thermal degradation. Meanwhile, in the composites, the interfacial interactions involving SiO_2_ and Si69 further restrict the diffusion and rearrangement of polymer chains during heating.

### 3.8. Electrical Conductivity of Ag/MVMPS Conductive Adhesive

**Figure 11:**
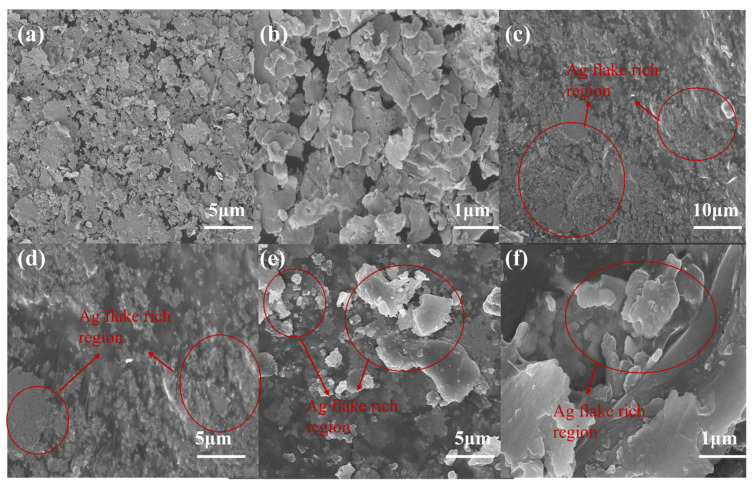
(**a**) low-magnification SEM image of flake silver powder; (**b**) high-magnification SEM image of flake silver powder; (**c**–**e**) low-magnification SEM image of Ag/MVMPS conductive adhesive; (**f**) high-magnification SEM image of Ag/MVMPS conductive adhesive.

During the preparation of conductive adhesive, the conductive silicone rubber becomes electrically conductive only when the volume fraction of the conductive filler reaches a certain critical level. The filler content at which conductivity first appears is referred to as the electrical percolation threshold [41]. Owing to their high aspect ratio and large contact area, flake silver powders can more readily overlap and form conductive networks, and therefore usually require a lower loading than spherical silver powders to achieve electrical conduction. SEM images of the flake silver powder are shown in Figure 11a,b. From the morphological observations, the silver powder exhibited a typical flake-like structure, with particles mainly in the form of thin platelets, irregular edges, and a certain degree of stacking and agglomeration. This morphology provides a structural basis for the subsequent formation of conductive contacts.

**Figure 12:**
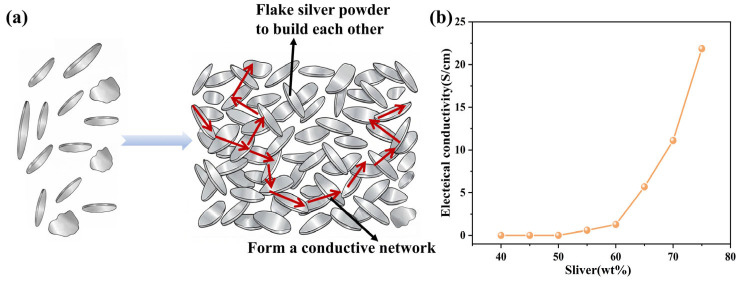
(**a**) schematic illustration of the formation of the conductive network of flake silver powder; (**b**) electrical conductivity of Ag/MVMPS conductive adhesive with different silver powder mass fractions.

To prepare the Ag/methyl vinyl phenyl silicone conductive adhesive, the electrical percolation threshold first had to be determined. Therefore, the relationship between the flake silver powder content and electrical conductivity was systematically investigated, and the results are shown in Figure 12b. When the flake silver powder content was below 50 wt%, the resistance of the conductive silicone rubber was too high to fall within the measurement range of the four-probe resistivity tester [42]. Once the silver powder content exceeded 50 wt%, the electrical conductivity of the conductive silicone rubber composite began to increase gradually, although the initial increase was relatively moderate. When the silver powder content reached 65 wt%, a pronounced increase in conductivity was observed, indicating that the system had approached and crossed the critical region for rapid formation of a conductive network. Accordingly, the electrical percolation threshold of this system can be considered to lie in the range of 60–65 wt%. Once this threshold was exceeded, a further increase in flake silver powder content significantly enhanced the conductivity of the material, leading to a substantial and continuous increase in electrical conductivity [43]. Therefore, a flake silver powder content of 65 wt% was selected as the conductive filler ratio for the preparation of the conductive silicone adhesive.

Figure 12a presents a schematic illustration of the evolution of the conductive network structure with the gradual addition of flake silver powder, clearly showing the process from isolated conductive domains to the formation of a continuous conductive network. The SEM image of Ag/vinyl phenyl silicon conductive adhesive in Figure 11 c–f can also reflect this point, and we mark the relatively enriched area/typical area of silver sheet in the figure. The electrical conductivity and piezoresistive response of Ag/MVMPS composites are closely related to the evolution of the conductive network formed by flake-like silver particles in the rubber matrix. At relatively high silver contents, adjacent silver flakes can come into contact or overlap with each other, forming continuous or semi-continuous conductive pathways and thus leading to contact conduction. In addition, some neighboring silver flakes are not in direct contact but are separated by nanoscale ultrathin polymer layers. In this case, electrons may undergo quantum tunneling through the polymer barrier [44]. Therefore, the conductivity of Ag/MVMPS composites can be attributed to the combined effect of contact conduction and tunneling conduction. Under applied strain, the interflake distance, contact area, and polymer barrier thickness change, which may cause reconstruction of the conductive pathways and variations in tunneling probability, ultimately resulting in changes in the resistance response.

In addition, the conductivity decay behavior of Ag/MVMPS conductive adhesives containing different mass fractions of flake silver powder was preliminarily investigated at room temperature and elevated temperature. Ag/MVMPS conductive adhesives containing 60 wt%, 65 wt%, and 70 wt% flake silver powder were placed in a constant-temperature oven and subjected to thermal treatment for 24 h and 72 h at 25 °C and 100 °C, respectively. The electrical conductivity of the conductive adhesives was then measured, and the conductivity decay rate was calculated. The results are summarized in Table 6. As can be seen from the data, the conductivity of this system remained relatively stable during storage at room temperature, whereas a more pronounced conductivity decay was observed under thermal aging at 100 °C. With increasing silver powder content, the conductivity decay rate became relatively lower. This can be attributed to the higher filler loading, which provided more redundant conductive pathways and thus a more stable conductive network. Under continuous thermal action, the contact state of silver flakes and the local conductive network in the Ag/MVMPS conductive layer may undergo microstructural reconstruction. Accordingly, a more significant conductivity attenuation occurs at 100 °C, especially under the aging condition of 72 h. The data indicate that the high-temperature conductive stability of this system still needs to be improved. In future work, the formulation will be further optimized, and needle-like conductive fillers may be considered to improve conductivity stability.

### 3.9. Sensing Performance of the Sandwich-Structured Strain Sensor

**Figure 13:**
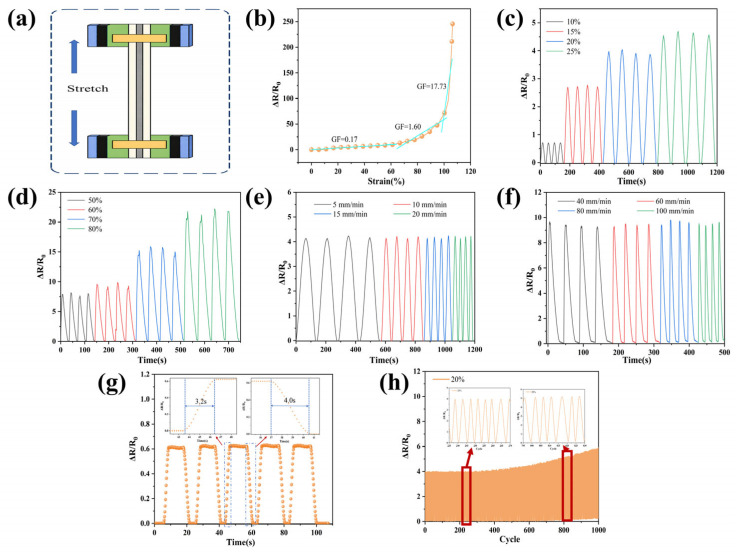
(**a**) sandwich sensor sensor tensile test equipment schematic diagram; (**b**) variation in ΔR/R_0_ as a function of strain; (**c**) strain-sensing response under 10–25% strain (increased by 5% in each cycle); (**d**) strain-sensing response under 50–80% strain (increased by 10% in each cycle); (**e**) strain-sensitive behavior at different frequencies under 20% strain; (**f**) strain-sensitive behavior at different frequencies under 60% strain; (**g**) sandwich sensor sensor response and recovery time (**h**) long-term tensile-release cycling stability test for 1000 cycles at 20% strain; the insets show the response signals over cycles 235–270 and 795–830.

Based on the above investigation of the methyl vinyl phenyl silicone rubber matrix and its composites, a phenyl silicone rubber/conductive adhesive/phenyl silicone rubber sandwich-structured sensor was fabricated in order to further explore its potential application in flexible electronics. Its strain-response behavior, rate adaptability, and cyclic stability were preliminarily evaluated, and the results are shown in Figure 13. The gauge factor (GF) was used to characterize the strain sensitivity and was defined as $GF=(\Delta R/R_{0})/\varepsilon$, and where $\Delta R=R-R_{0}$, and R and $R_{0}$ represent the resistance of the sample during loading and its initial resistance, respectively, while ε is the corresponding strain value [45]. The relationship between GF and strain for the sandwich-structured strain sensor is shown in Figure 13a. It can be seen that the GF generally increased with increasing strain and exhibited an obvious nonlinear variation. This is because the spacing between silver flakes increases during stretching, causing separation of partial conductive contacts. When the strain is released, part of the conductive network can recover. Nevertheless, under repeated strain cycling, slight sliding and rearrangement of silver flakes may occur, triggering the reconstruction of local conductive networks, which consequently results in certain differences or hysteresis of electrical response in different cycles. To more comprehensively evaluate the strain-sensing performance of the sandwich-structured sensor, its electrical response under different strain levels and loading rates was measured, and the results are shown in Figure 13b-e. The results demonstrated that the device could output clear and stable periodic resistance-response signals over a strain range of 10–80%, and the response intensity increased significantly with increasing strain, indicating good strain discrimination capability. The results under different loading rates showed that, at the same strain level, the peak response of the sensor changed only slightly, and the cyclic waveform remained complete, indicating that its electrical output was mainly governed by the deformation level and that the device possessed good dynamic response capability and rate adaptability. At the same time, we use cyclic tensile test at 10% strain, the response time is 3.2 s, the recovery time is 4.0 s, as shown in Figure 13g. In addition, after 1000 cycles at 20% strain, the device still maintains a continuous and identifiable response signal, as shown in Figure 13f. The enlarged local views further showed that the single-cycle response profile retained good readability and repeatability at different stages of the cyclic test. At the same time, we also observed that as the number of cycles increases, there is a slight drift in the response peak after 800 cycles, but it still maintains a stable periodic output. This signal drift is mainly attributed to the viscoelastic relaxation of the silicone rubber matrix, the residual deformation during cyclic stretching, and the gradual reconstruction of the flake silver conductive network. These preliminary results indicate that the sandwich-structured sensor has good application potential in flexible strain sensing.

According to the literature, rGO/GO@CA/TPU polyurethane composite sensors have been used for subtle deformation detection, showing a gauge factor (GF) of approximately 3.006, while their effective sensing range is mainly concentrated in the low-strain region of 0–10% [46]. MXene/TPU/PAN sensors can achieve a GF of 9.69 with a strain range of 0–80% [47]. Polyurethane-based strain sensors generally exhibit high sensitivity and a relatively wide response range; however, these materials still have certain limitations in terms of thermal stability. Under high-temperature conditions, polyurethane is prone to mechanical and electrical degradation, which restricts its applicability in harsh environments. In contrast, silicon-based strain sensors, such as those based on PDMS or silicone rubber, typically exhibit better heat resistance and environmental stability. For example, CNT/PDMS sensors have been reported to show a maximum GF of approximately 10 and a response range of up to 60% [48]. CNT/CB/silicone rubber composites have also been used for strain sensors, achieving a response range of 100% and exhibiting stable and durable electrical responses [33]. In addition, PDMS foam-based sensors exhibit a GF of approximately 2.25 over a strain range of 0–80% [49]. The sandwich-structured sensor developed in this work shows a GF of approximately 1.7 in the 60–90% strain region, which increases to 11.4 at strains above 90%. Meanwhile, it can operate over a broad strain range of 0–105%, demonstrating promising application potential.

## 4. Conclusions

In this study, MVMPS with high vinyl and phenyl contents was synthesized from P_3_ V_3_, and D_4_ via anionic ring-opening polymerization. The obtained MVMPS exhibited a vinyl content of 24.44% and a phenyl content of 25.19%. It was further condensation-modified with KH-570 to obtain C-MVMPS. Using fumed silica as the reinforcing filler, a phenyl silicone rubber composite system synergistically regulated by SiO_2_ and Si69 was successfully constructed. The results showed that Si69 played dual roles in the system, namely, coupling and crosslinking regulation, which effectively improved filler dispersion and interfacial interaction and enhanced the uniformity of the composite network structure. Among the investigated formulations, the composite containing 3 phr Si69 and 30 phr SiO_2_ exhibited the best overall performance, including excellent heat resistance, with a T_5%_ of up to 367.14 °C and a T_10%_ of 529.6 °C, good mechanical strength, with a tensile strength of 4.69 MPa, and good toughness, with an elongation at break of 206.28%.In the conductive system, the electrical percolation threshold of flake silver powder was determined to lie within the range of 60–65 wt%, and 65 wt% was selected as the formulation for the conductive sensing layer. Based on this, a silicone rubber/Ag-MVMPS conductive adhesive/silicone rubber sandwich-structured sensor was further fabricated. The sandwich-structured sensor exhibited good repeatability and stability under different strain levels and loading rates and still maintained a clear periodic response after 1000 cycles at 20% strain. These results indicate that the sandwich-structured sensor possesses basic strain-sensing capability and a certain degree of cyclic stability, demonstrating promising application potential in the field of flexible strain sensing.

## Figures and Tables

**Table 1:** Effect of different catalysts on the molecular weight of silica gel.

Catalyst	Material Content/mol	Mn ^a^ (g/mol)	Mw ^b^ (g/mol)	Room Temperature State
	D_4_/V_3_/P_3_			
Sodium ethoxide	100/50/50	82,898	337,847	Solid
Stannous octoate	100/50/50	50,076	17,545	Viscous liquid
Tetramethylammonium hydroxide	100/50/50	170,449	567,197	Solid

^a^ Number: average molecular weight; ^b^ Weight: average molecular weight.

**Table 2:** MVMPS group content.

Product	Vinyl Content		Phenyl Content	
	Theoretical	Calculated	Theoretical	Calculated
MVMPS	25%	24.44%	25%	25.19%

**Table 3:** Mechanical properties of MVMPS composites with different types and fractions of vulcanizing agents.

Vulcanizing Agent-(phr ^a^)	Tensile Strength/MPa	Elongation at Break/%
DCP ^b^-0.5 ^c^	2.96	49
DCP-1	3.67	54.32
DCP-2	Light touch cracking	/
DHBP-0.5	2.21	52.94
DHBP-1	2.88	74.74
DHBP-2	Light touch cracking	/

^a^ Parts per hundred of rubber; ^b^ Types of vulcanizing agents; ^c^ Different dosages of vulcanizers.

**Table 4:** Mechanical properties of MVMPS composites with different Si69 content.

Vulcanizing Agent-(phr ^a^)	Tensile Strength/MPa	Elongation at Break/%
Si69 ^b^-1 ^c^	2.69	113.3
Si69-2	3.35	141.52
Si69-3	4.69	206.28
Si69-4	5.26	101.84

^a^ Parts per hundred of rubber; ^b^ Types of vulcanizing agents; ^c^ Different dosages of vulcanizers.

**Table 5:** Vulcanization characteristics of SiO_2_/MVMPS composites.

Group-(phr ^a^)	t_c10_ ^d^/min:s	t_c90_ ^e^/min:s	M_L_ ^f^/d·Nm	M_H_ ^g^/dN·m	M_H_-M_L_(ΔM) ^h^/dN·m
Si69 ^b^-1 ^c^	1:48	23:20	0.76	2.45	1.69
Si69-2	1:26	23:21	1.17	2.7	1.53
Si69-3	1:36	23:40	0.71	3.88	3.17
Si69-4	1:53	23:54	2.57	6.51	3.94
SiO_2_-10	1:30	23:56	0.73	3.1	2.37
SiO_2_-20	1:23	23:15	0.8	3.38	2.58
SiO_2_-30	1:36	23:40	0.71	3.88	3.17
SiO_2_-40	1:16	23:55	7.93	18.73	10.8

^a^ Parts per hundred of rubber; ^b^ Types of vulcanizing agents; ^c^ Different dosages of vulcanizers; ^d^ Scorch time; ^e^ Optimum curing time; ^f^ Minimum torque; ^g^ Maximum torque; ^h^ Torque difference.

**Table 6:** Ag/MVMPS conductive adhesive conductivity(S/cm) of the attenuation rate.

Content	25 °C for 24 h	25 °C for 72 h	100 °C for 24 h	100 °C for 72 h
60 wt%	1.72% ^a^	6.79%	17.7%	33.25%
65 wt%	1.44%	4.79%	14.82%	28.70%
70 wt%	1.62%	5.40%	12.60%	25.50%

^a^ Relative to the measured value at room temperature.

## Data Availability

The original contributions presented in this study are included in the article.
